# Spinal cord infarction secondary to pulmonary embolism-induced cardiac arrest: a case report

**DOI:** 10.1186/s12871-022-01820-4

**Published:** 2022-08-31

**Authors:** Jianfei Xu, Xiaoyang Zhou, Zhicheng Liu, Zhaojun Xu

**Affiliations:** 1 Department of Intensive Care Medicine, HwaMei Hospital, University of Chinese Academy of Sciences, Ningbo, 315000 Zhejiang China; 2 Department of Intensive Care Medicine, Xiangshan Red Cross Taiwan Compatriot Hospital Medical and Health Group, Ningbo, 315000 Zhejiang China

**Keywords:** Spinal cord infarction, Cardiac arrest, Pulmonary embolism, Magnetic resonance imaging

## Abstract

**Background:**

Pulmonary embolism is a common cause of cardiac arrest. Pulmonary embolism-induced cardiac arrest typically suffers from ischemic injuries to various organs, including the central nervous system. However, spinal cord infarction is a rare complication of pulmonary embolism-induced cardiac arrest. At present, there is no case report on the occurrence of spinal cord infarction secondary to pulmonary embolism-induced cardiac arrest without accompanied cerebral complications.

**Case presentation:**

A 72-year-old woman with dyspnea and chest tightness was admitted to the emergency room. Cardiac arrest occurred within a short period after admission. Subsequent computed tomographic pulmonary angiography revealed multiple pulmonary thromboses, which were highly suspected to be the cause of cardiac arrest. Thrombolytic therapy with alteplase was given after the return of spontaneous circulation. Unfortunately, she was found to be paraplegic in both lower extremities after regaining consciousness. Spinal cord infarction was confirmed by thoracic magnetic resonance imaging.

**Conclusions:**

Despite receiving high-quality cardiopulmonary resuscitation, patients with cardiac arrest are at high risk of ischemic injury to the central nervous system. After the recovery of consciousness, clinicians should pay more attention to preclude the possibility of spinal cord infarction.

## Introduction

Pulmonary embolism (PE) is a relatively common vascular embolic disease. The clinical presentations of PE are variable, ranging from slight dyspnea in mild cases to unstable hemodynamics or even life-threatening cardiac arrest (CA) in severe cases [1]. Once peoples suffer from PE-induced CA, the rate of ischemic injury of organs increases with prolonged cardiopulmonary resuscitation (CPR) [2]. Spinal cord infarction (SCI) is a rare complication as compared to cerebral infarction, although they share a similar pathophysiological mechanism [3]. The common causes of SCI include thoracoabdominal aortic surgery, trauma, vascular diseases (e.g., thromboembolic and inflammatory diseases), and systemic hypoxic-ischemic events such as CA or severe hypotension [4]. Although a wide spectrum of neurologic complications may follow prolonged CPR, SCI caused by PE-induced CA without accompanying cerebral complications has never been reported. Herein, the authors present a rare case of elderly patients who developed acute SCI after prolonged CPR to resuscitate PE induced-CA.

## Case report

**Fig. 1:**
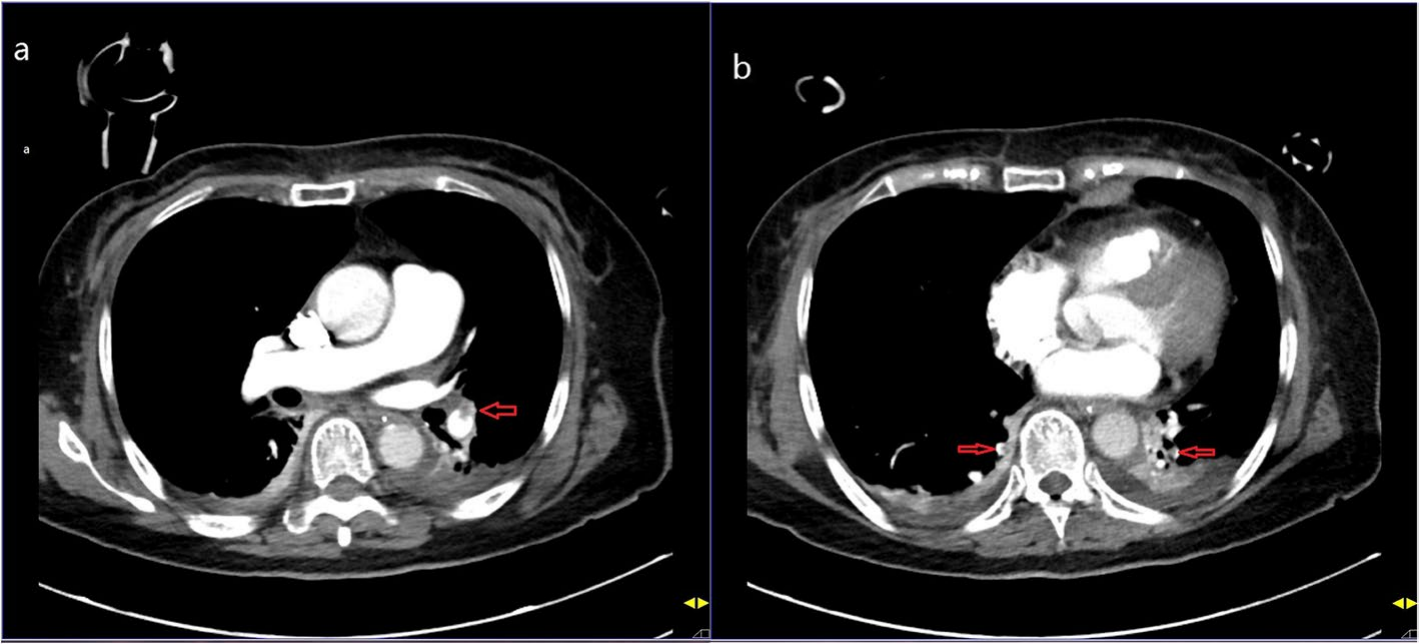
Computed tomography pulmonary angiography shows extensive thrombus burden in segmental branches of pulmonary artery. **a**: thrombus (red arrow) in left lower pulmonary artery trunk; **b**: thrombi (red arrows) in posterior basal segment of both lower lobes

A 72-year-old woman without a history of any coexisting disease was admitted to the emergency room with a complaint of dyspnea and chest tightness. Physical examination showed obvious cyanosis of lips; vital signs indicated an unstable hemodynamic status, with a heart rate of 102 beats/min, a respiratory rate of 25 beats/min, and blood pressure of 88/51 mmHg, finger oxygen saturation of 61%, and body temperature of 36.0 °C. The initial electrocardiogram at admission suggested sinus tachycardia, nonspecific intraventricular block, abnormal q waves (leads III, V1), ST-segment depression (leads V2, V3, V4), T wave inversion (leads II, III, avF, V1, V2, V3, V4), and ECG axis right deviation. CA occurred 10 minutes later, and high-quality CPR was performed immediately. The blood test revealed a high level of D-Dimer of 8051 ng/ml. After 60 minutes of high-quality CPR, she returned to spontaneous circulation and was transferred to the intensive care unit due to unstable hemodynamics. Subsequent ultrasonography examination revealed an intermuscular vein thrombosis (4.3 mm) in the right lower extremity. Computed tomographic pulmonary angiography (CTPA) revealed a thrombus in the left lower pulmonary artery trunk and multiple small thrombi in the pulmonary artery branches (Fig. 1). Thus, PE was highly suspected as the cause of CA. Then, the patient received thrombolytic therapy with 50 mg of recombinant tissue plasminogen activator (alteplase).

**Fig. 2:**
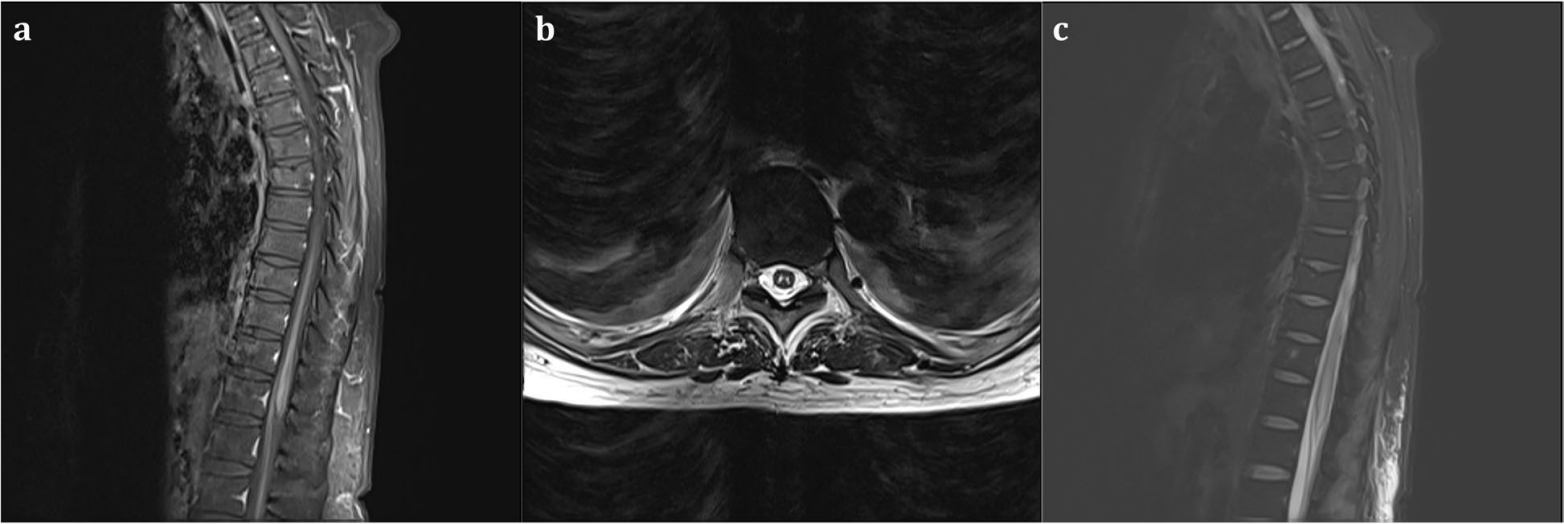
Thoracic spinal cord MRI. Sagittal T1-weighted MRI shows signal enhancement in the gray matter of the spinal cord (**a**); Axial T2-weighted MRI shows increased signal intensity in the gray matter segment of the spinal cord (**b**); Sagittal fat-suppressed sequence MRI shows enhanced signals of spinal cord swelling (**c**)

Four days later, she regained consciousness and was able to perform commanded movements. Unfortunately, she was diagnosed with paraplegia. Physical examination showed that Glasgow Coma Scale (GCS) score was E4 + VT + M6; the muscle strength of both upper limbs was grade 5 with normal sensation, and upper limb deep and superficial reflexes were present, but the muscle strength of both lower extremities was grade 0, and pain and temperature sensation disappeared. Deep tendon reflexes were absent at the bilateral lower limbs. However, the sense of touch and proprioception existed below the T9 level of the spinal cord. Thoracic spinal cord 3.0 T contrast-enhanced magnetic resonance imaging (MRI) (Fig. 2) indicates T7 - T12 thoracic spinal cord swelling with abnormal signal, showing ischemia-like changes, “pencil-like” sign in sagittal view, and “eagle eye” sign in the cross-sectional scan. The neurologist’s consultation concluded that she was diagnosed with spinal cord infarction, which was consistent with anterior spinal artery syndrome. At discharge from the hospital after 23 days of treatment, her neurological condition deteriorated; she lost bowel and bladder sensation.

## Discussion and conclusion

In this article, we reported a rare case of SCI secondary to PE-induced CA. Although SCI is an uncommon complication of CA, this case report shows an important implication for clinicians that one should pay more attention to muscle strength of extremities to early identify SCI after the recovery of consciousness in CA patients who have received high-quality CPR.

To the best of our knowledge, this is the first case reporting SCI caused by PE-induced CA. SCI is a quite rare but catastrophic disease that accounts for only 1 to 2% of all ischemic strokes and 5 to 8% of acute myelopathies [5, 6]. The mechanism of spinal cord ischemia is multifactorial and involves aortic surgery, spine compression, vascular and thromboembolic diseases, and systemic hypoxic-ischemic events [4, 7]. Blood to the anterior two-third of the spinal cord is supplied by the anterior spinal artery. The anterior spinal artery has a less efficient collateral supply and is thus more vulnerable to ischemia. Ischemia and hypoxia are prone to occur when systemic perfusion is insufficient, especially for prolonged hypoperfusion [4]. In this case, we infer that the most probable reason for the occurrence of SCI is systemic hypoperfusion under prolonged CPR after PE-induced CA. Although the patient received a timely high-quality CPR, the long period of CA (60 minutes) inevitably led to prolonged systemic hypoperfusion, which is a well-known predisposing factor of central nervous system ischemia, finally causing ischemic infarction of the spinal cord. Although deep venous thrombosis in the right lower extremity was found by vascular ultrasonography examination, which could be associated with PE, paradoxical embolism is very infrequent to occur to generate spinal arterial occlusion. Spinal cord occlusive infarction attributing to atherosclerotic material detaching from the aorta walls or its branches is theoretically possible during CPR, but it is also rarely occurred [8].

The diagnosis of SCI is typically difficult and primarily depends on clinical manifestation and spine MRI examination, while highlighting noninflammatory findings in cerebrospinal fluid tests [9]. In patients who suffered CA and successful CPR, the diagnosis of SCI relies on the recovery of consciousness. Therefore, the diagnostic rate of SCI may be underestimated in patients who suffered from CA because regained consciousness is not always obtained. A retrospective study found that 46% of patients who died of CA or severe hypotension had spinal cord ischemic lesions [10]. In this case, consciousness was retained on the 4th day after the return of spontaneous circulation (ROSC), and the patient presented with flaccid paraplegia, loss of pain and temperature sensation, and presence of touch and proprioception below T8 - T9, which is consistent with previous reports [10, 11]. Moreover, enhanced spine MRI (Fig. 2) in this case furtherly supports the diagnosis of SCI. The prognosis of SCI is variable in CA patients who have undergone prolonged CPR. There are kinds of literature reporting that one case died of multiorgan failure on the 38th day after ROSC [11], one case showed no improvement in neurologic function at 2-year follow-up [12], and one case partially recovered [13]. In this case, the patient’s neurological symptoms deteriorated at the time of discharging from the hospital; she lost bowel and bladder sensation.

This case report shows several important implications for clinicians. First, a survivor of CA undergoing prolonged CPR may only develop SCI without cerebral ischemia. Thus, one should pay more attention to peripheral neurological evaluation to assess possible lesions in any part of the nervous system and early identify SCI after the recovery of consciousness in CA patients. Second, enhanced spine MRI is an effective and reliable diagnostic tool for diagnosing spinal cord ischemia and should be examined early in patients with suspicious clinical presentation of SCI. Lastly, high-quality CPR is critically important for ROSC; however, it cannot ensure the sufficient perfusion of the central nervous system.

In conclusion, patients with CA are at high risk of ischemic injury to the central nervous system, despite receiving high-quality CPR. After the recovery of consciousness, clinicians should pay more attention to preclude the possibility of SCI.

## Data Availability

Data sharing not applicable to this article as no datasets were generated or analyzed during the current study.

## Abbreviations

PE: Pulmonary embolism
CA: Cardiac arrest
CPR: Cardiopulmonary resuscitation
SCI: Spinal cord infarction
CTPA: Computed tomographic pulmonary angiography
MRI: Magnetic resonance imaging
ROSC: Return of spontaneous circulation
